# Administration of *M. leprae* Hsp65 Interferes with the Murine Lupus Progression

**DOI:** 10.1371/journal.pone.0003025

**Published:** 2008-08-21

**Authors:** Eliana B. Marengo, Luciana V. de Moraes, Marcella Faria, Beatriz L. Fernandes, Luciana V. Carvalho, Denise V. Tambourgi, Luiz V. Rizzo, Fernanda C. V. Portaro, Antônio Carlos M. Camargo, Osvaldo A. Sant'Anna

**Affiliations:** 1 Immunochemistry Laboratory, Instituto Butantan, São Paulo, Brazil; 2 Clinical Immunology Laboratory, Instituto de Ciências Biomédicas, University of São Paulo, São Paulo, Brazil; 3 Center for Applied Toxinology – CAT/CEPID, São Paulo, Brazil; University of Sheffield, United Kingdom

## Abstract

The heat shock protein [Hsp] family guides several steps during protein synthesis, are abundant in prokaryotic and eukaryotic cells, and are highly conserved during evolution. The Hsp60 family is involved in assembly and transport of proteins, and is expressed at very high levels during autoimmunity or autoinflammatory phenomena. Here, the pathophysiological role of the wild type [WT] and the point mutated K^409^A recombinant Hsp65 of *M. leprae* in an animal model of Systemic Lupus Erythematosus [SLE] was evaluated *in vivo* using the genetically homogeneous [NZBxNZW]F_1_ mice. Anti-DNA and anti-Hsp65 antibodies responsiveness was individually measured during the animal's life span, and the mean survival time [MST] was determined. The treatment with WT abbreviates the MST in 46%, when compared to non-treated mice [*p*<0.001]. An increase in the IgG2a/IgG1 anti-DNA antibodies ratio was also observed in animals injected with the WT Hsp65. Incubation of BALB/c macrophages with F_1_ serum from WT treated mice resulted in acute cell necrosis; treatment of these cells with serum from K^409^A treated mice did not cause any toxic effect. Moreover, the involvement of WT correlates with age and is dose-dependent. Our data suggest that Hsp65 may be a central molecule intervening in the progression of the SLE, and that the point mutated K^409^A recombinant immunogenic molecule, that counteracts the deleterious effect of WT, may act mitigating and delaying the development of SLE in treated mice. This study gives new insights into the general biological role of Hsp and the significant impact of environmental factors during the pathogenesis of this autoimmune process.

## Introduction

The Hsp60 guides essential activities for cell homeostasis being highly conserved among organisms; in stress or inflammation conditions, they increase 4 to 5-fold in the cell subsequently undergoing autolysis returning to basal levels [1]. The distinct Hsp classes show extensive amino acid sequence similarities, from microbial to mammalian. The mycobacterium 65 kDa, a member of the Hsp60 family, shows an approximately 55% similarity/identity with human Hsp60 [2], [3]. The difference between Hsp60 and Hsp65 is striking in regions containing epitopes recognized by T cells of the vertebrate host and the cross-reactivity between these determinants is due to conserved regions across all organisms.

Stress proteins of a wide variety of microorganisms have been found to stimulate an immune response in vertebrates [4], [5], but the evolutionary consequences of this recognition are poorly understood. Published data show that Hsp65 is one of the major target for the immune response to pathogens [6], [7]. The major sequence similarity among species renders the Hsp65 a potential inducer of immune responses to host self molecules that may lead to autoimmune phenomena. Due to high similarity inter-species in their sequence, it is suggested from a process of molecular mimicry, the participation of the Hsp60 in the modulation and etiology or pathogenesis of autoimmunities [8]–[11]. Pockley et al [12] indicated the presence of antibodies for Hsp70 family in healthy individuals, such as that of Stephanou et al [13] showing significant increase of anti-Hsp90 antibody titers in patients with SLE compared to healthy individuals. There are several studies that attempt to establish the relation between Hsp60 and inflammatory and proliferative responses. High anti-Hsp60/65 antibody titers were not restricted to disease patients and could also be detected during aging [12], [14]–[16].

Recently, our group described that the recombinant Hsp65 from *Mycobacterium leprae* displays proteolytic activity towards oligopeptides. The amino acid sequence alignment of the *M. leprae* Hsp65 with the *Escherichia coli* HslVU protease suggested two putative threonine catalytic groups, one in the N-domain [Thr^136^, Lys^168^, Tyr^264^] and the other in the C-domain [Thr^375^, Lys^409^, Ser^502^]. Mutagenesis studies showed that these amino acid residues at the C-domain form the catalytic group that carries out the main proteolytic activity of this molecule [17].

Systemic lupus erythematosus [SLE] is the prototypic autoimmune disease, influenced by a combination of genetic and environmental factors and characterized by a marked loss of tolerance to self antigens such as DNA, RNA and other nuclear factors, followed by immune-mediated injury to multiple organs [18]. The New Zealand Black [H–2*^d2^*] × New Zealand White [H–2*^z^*] [NZBxNZW]F_1_ hybrid mouse is genetically predisposed to develop autoimmune disease, which resembles SLE in humans. The evolution of this chronic inflammatory disease is characterized by an abnormal polyclonal B cell activation with a elevated production of antinuclear antibodies that include anti-double-stranded DNA, lymphadenopathy, arthritis, hemolytic anemia, vasculitis and glomerulonephritis [19], [20]. DNA plays a central role in the pathogenesis of SLE, serving as a target antigen of auto–antibodies as well as a major component of immune complexes [21], [22]. Whereas the origin of the autoantibodies in SLE has received intense investigation, the mechanisms involved in increased anti-DNA titers are not well understood. It has been suggested that accelerated monocytes/macrophages apoptosis in SLE contributes to impaired clearance of apoptotic cells and increases apoptotic material [23], [24]. Similarly to humans, the experimental disease is most frequent and severe in female mice and the pathologic features appear when these animals are around 6-month-old and they usually evolve to death around 10–12-month of age [25]–[27].

Motivated by the Ilya Prigogine theoretical proposition [28], [29] that living systems achieve ordered state from relatively disorganized configurations by assembling a new type of dynamic state defined as dissipative structures. In this study the purpose was to establish an original approach whereas disequilibrium is induced by passive administration of homologous proteins. The attempts of the system to reestablish an equilibrium situation seem severely compromised in [NZBxNZW]F_1_ autoimmunity prone mice. Thus, the putative pathophysiological role of the wild type [WT] and the one point mutated K^409^A recombinant Hsp65 [rHsp65] of *M. leprae* in SLE was extensively evaluated in genetically homogeneous [NZBxNZW]F_1_ mice treated at distinct ages. Clinical signs were analyzed, including development of ascites, pile erection, lethargy, anorexia, as well as the mean survival time. In addition, the quantitative phenotypic traits of anti-DNA and anti-Hsp65 antibody responsiveness were individually determined during one year of life span. The *in vitro* induction of apoptosis/necrosis by serum from WT or K^409^A rHsp65 treated F_1_ mice were also evaluated in normal macrophages. The results suggest the involvement of the Hsp65 as a central molecule intervening in the foundation and progression of the SLE syndrome. The relation between phenotypes in untreated and WT rHsp65 treated individuals, as the K^409^A mutant employed, gave new insights into the general biological role of Hsp molecules, as potential neutralizers of the great impact of environmental factors on SLE.

## Results

### WT rHsp65 treatment accelerates death in SLE

**Figure 1:**
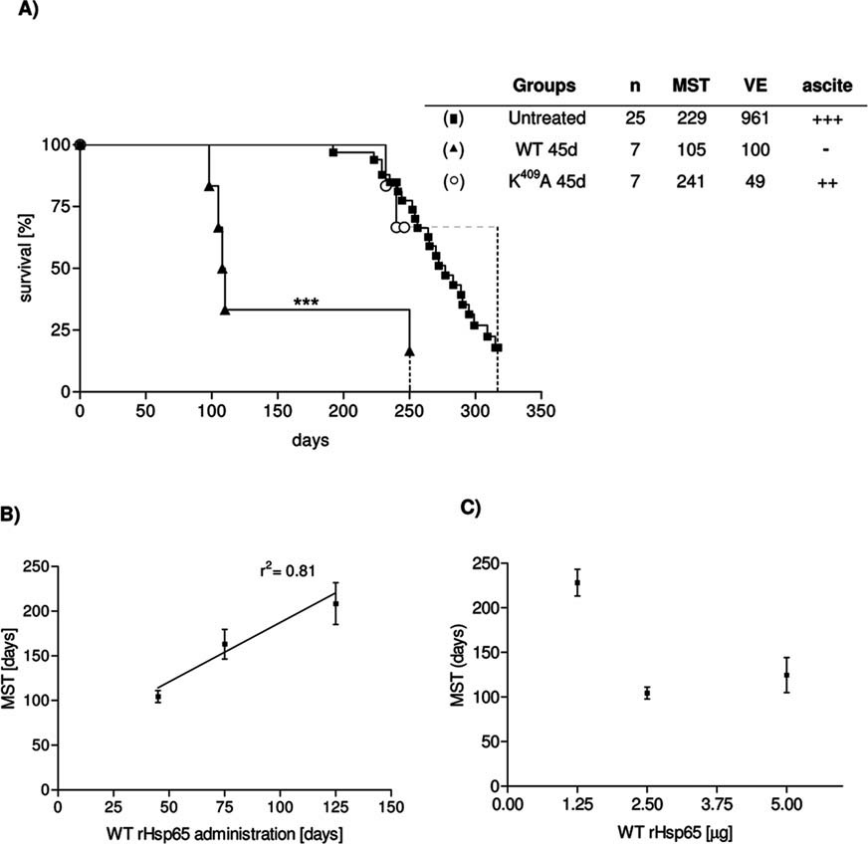
Effect of the WT and K^409^A rHsp65 treatment on SLE. Effect of the WT rHsp65 administration on survival time [A]. Female [NZBxNZW]F_1_ mice were treated or not [▪] with a single dose [2.5 µg] of WT [▴] or K^409^A [○]rHsp65 at 45 days of age. Age-dependence effect of the WT rHsp65 treatment on MST [B]. Dose-dependence of WT rHsp65 on F_1_ MST [C]. MST [Mean survival time; days]; VE [environmental variance]; Ascite: development of ascite [+++: high incidence; -: not development]. Untreated group has been the reference group for log-rank analysis; ***p<0.001. Results are representative of 2 independent experiments.

To determine the effect of *M. leprae* rHsp65 on the development of autoimmunity, 45 days-old [NZBxNZW]F_1_ mice were injected with a single dose of the WT rHsp65 or with the point mutated K^409^A rHsp65, and monitored until death, and compared to untreated mice. Figure 1A shows that death was significantly accelerated in mice treated with WT rHsp65. Their mean survival time [MST] was 105 days, as compared to 229 days of untreated mice, and 241 days of the K^409^A-treated group. Untreated and K^409^A-treated animals presented the typical clinical signs of murine lupus: anemia, proteinuria, including progressive ascites starting at day 180. We also evaluated if the Hsp injection would have an impact on survival of older mice by administering a single dose of WT rHsp65 at ages 75 or 125 days. Figure 1B shows that survival progressively increased in the late injected groups, presenting MST of 166 and 209 days, respectively. Noteworthy, none of the treated animals presented ascites, considered a primary characteristic of classical SLE in this murine model [19], [20]. It becomes evident that the earlier the treatment with WT rHsp65, the more precocious is death, and, as shown in figure 1B, a positive correlation of 81% [*p*<0.05] between the MST and age, during WT rHsp65 administration, was determined.

In this series of experiments the impact of environmental factors in SLE is also clearly evidenced: the environmental variance [VE] of 961 days for the untreated group was significantly higher than that for the WT [100 days] or K^409^A [49 days] treated mice at 45 days of age. Interestingly, for this last group, although the MST had been almost the same as that of the untreated mice, the survival was manifestly prolonged.

### Analysis of the dose-effect response

As shown in Figure 1A, animals treated with 2.5 µg of WT rHsp65 at the age of 45 days presented a MST of 105 days. These values were taken as a reference to be compared to 45 days-old animals receiving 1.25 µg or 5.0 µg of WT rHsp65. Figure 1C shows that mice treated with 1.25 µg display a MST of 221 days, whereas treatment with 5.0 µg showed a significantly shorter MST of 114 days.

### WT Hsp65 treatment enhances anti-DNA antibody response but has no effect on glomerulonephritis

**Figure 2:**
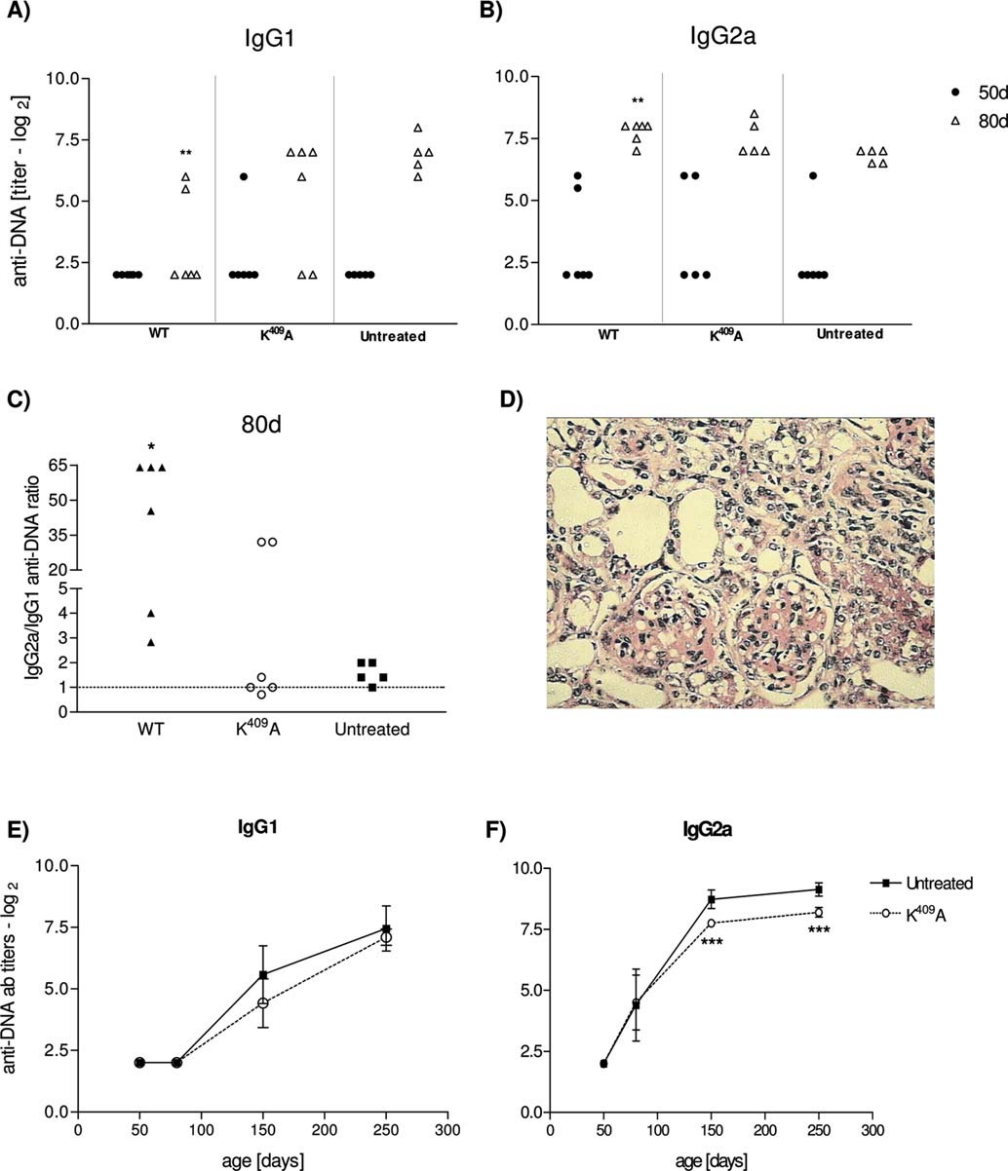
WT and K^409^A rHsp65 treatment in anti-DNA antibody response. Anti-DNA IgG1 [A] and IgG2a [B] isotypes antibody production and the IgG2a/IgG1 antibody ratio [C] in the serum of treated and untreated [NZBxNZW]F_1_ mice [n = 5–6/group]. Representative renal histological appearance from 4 to 5-month-old F_1_ mice; light microscopy 100× [D]. Time-course production of anti-DNA IgG1 [E] and IgG2a [F] antibodies in untreated and K^409^A mice. Untreated group has been the reference group for unpaired *t*-test analysis; *p<0.05; **p<0.01; ***p<0.001. Results are representative of 3 independent experiments.

In order to evaluate how the treatment with WT Hsp65 accelerates disease progression, the anti-DNA autoantibody production and glomerulonephritis incidence were analyzed in treated and untreated mice. IgG1 and IgG2a anti–DNA antibody titers were determined, when animals were 50 and 80 days-old. The anti-DNA IgG1 antibodies reveal a significant decrease in WT treated mice, as compared to untreated animals [Figure 2A]. Figure 2B shows an increase of anti-DNA IgG2a antibodies in 80 days-old WT treated mice, as compared to untreated ones [*p*<0.001], The ratio of IgG2a/IgG1 anti-DNA in animals treated with the WT protein was approximately 4 and 25 times increased, as compared to the IgG2a/IgG1 antibody ratio in the K^409^A treated and untreated groups, respectively [Figure 2C]. The anti-DNA IgG1 and IgG2a isotypes production in K^409^A treated mice behaved similarly to the untreated group, maintaining equal amounts of IgG isotypes along the life [Figures 2E and 2F]. Regarding glomerulonephritis, there was no increase of cellular infiltration, nor inflammation of the membrane, nor significant glomerular and interstitial changes in WT treated animals, as compared to the untreated and to K^409^A treated mice [Figure 2D]. Moreover, seric urea levels were kept normal and unchanged among experimental groups [data not shown].

### Anti-rHsp65 isotypes and cytokine production

**Figure 3:**
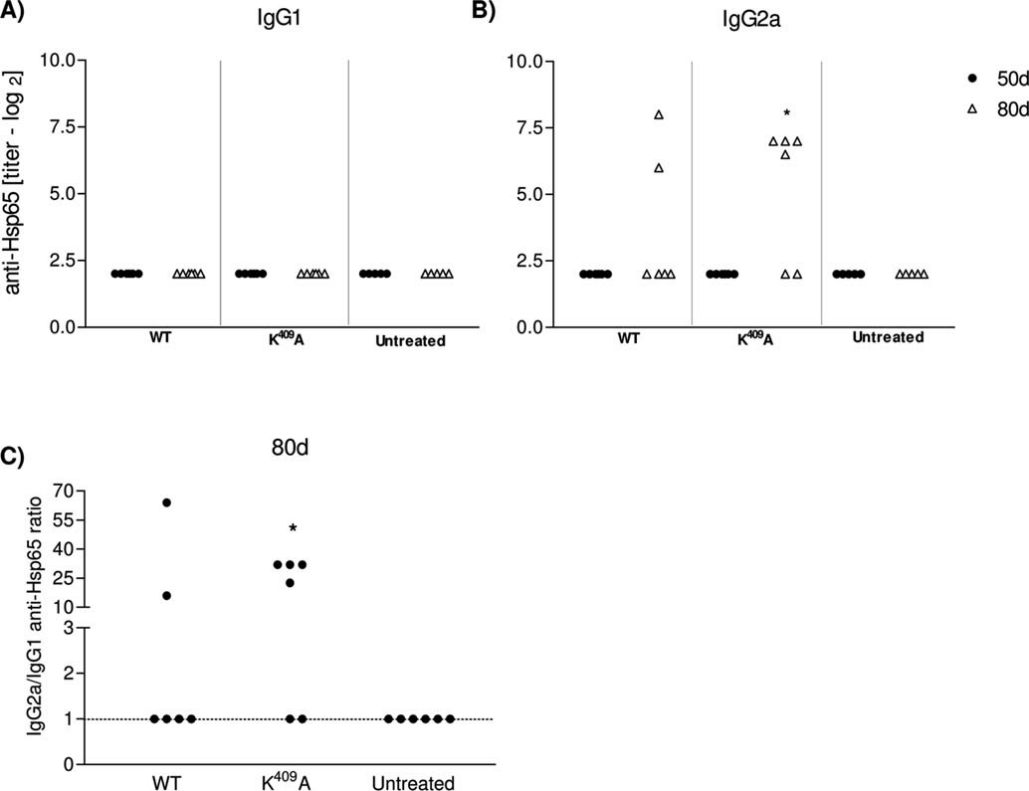
WT and K^409^A rHsp65 treatment in anti-Hsp65 antibody response. Anti-Hsp65 IgG1 [A] and IgG2a [B] antibody production, respectively, in the serum of treated and untreated [NZBxNZW]F_1_ mice of 50 and 80 days-old [n = 5–6/group]. IgG2a/IgG1 anti-Hsp65 antibody ratio [C] in WT, K^409^A and untreated groups in 80 days-old F_1_ mice. Antibodies were measured by isotype-specific ELISA in serum of 50 and 80 days-old mice. Untreated group has been the reference group for unpaired *t*-test analysis; *p<0.05. Results are representative of 3 independent experiments.

Anti-Hsp65 antibodies were determined in WT rHsp65 or K^409^A treated mice at 45 day of age, and in untreated groups. Individual antibody titers were measured in 50 and 80 days-old mice. Anti-Hsp65 IgG1 antibodies were not detected in any of the groups [Figure 3A], whereas IgG2a antibodies were observed in both, WT and K^409^A treated animals [Figure 3B]. There was a 3 fold increase in IgG2a antibody production in K^409^A, as compared to WT treated animals at day 80, and the IgG2a/IgG1 ratio was 10 times increased, when compared to untreated mice [Figure 3C]. During life the time-course of anti-Hsp65 IgG2a isotype response in K^409^A treated mice showed increased titers, as compared to untreated animals, whereas IgG1 was not detected, and behaved similarly to the untreated group [data not shown].

Anti-Hsp65 antibody production was also evaluated in mice genetically selected for high antibody responsiveness – H_III_ line – after subcutaneous immunization with 5 µg of WT or K^409^A rHsp65, emulsified with Incomplete Freund's Adjuvant. Anti-Hsp65 IgG antibody titers of approximately 6log_2_ were obtained from day 7 and achieving 7-8log_2_ at the day 30 in H_III_ mice that received one dose of K^409^A protein, whereas very low IgG antibody titers [about 4.5log_2_] to anti-Hsp65 in WT immunized mice could be detected, only in a secondary response.

**Figure 4:**
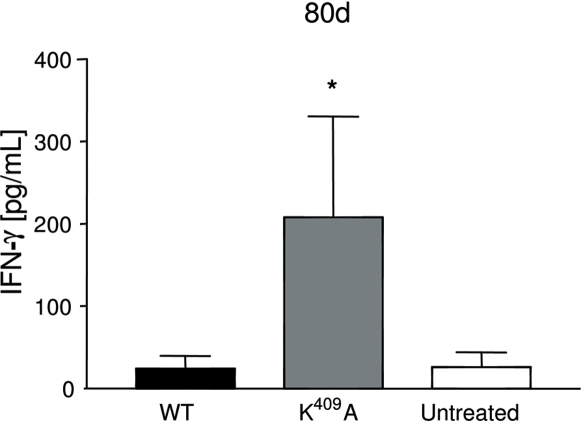
Enhanced interferon-γ production in the serum of K^409^A treated [NZBxNZW]F_1_ mice. IFN-γ was measured in the serum of 80 days-old WT or K^409^A treated or untreated [NZBxNZW]F_1_ mice [n = 5–6/group] by ELISA. Untreated group has been the reference group for unpaired *t*-test analysis; *p<0.05. Results are representative of 2 independent experiments.

As shown in Figure 4, a significant difference of 3–4 fold higher IFN-γ-levels was observed in the K^409^A group [*p*<0.05], when compared to the WT and untreated groups. The TNF-α and IL-6 levels were produced equally in all groups [data not shown]. This isotype profile and IFN-γ responses suggest that the K^409^A rHsp65 could have an immunomodulatory role in this experimental model.

### Analysis of apoptosis/ cell necrosis

**Figure 5:**
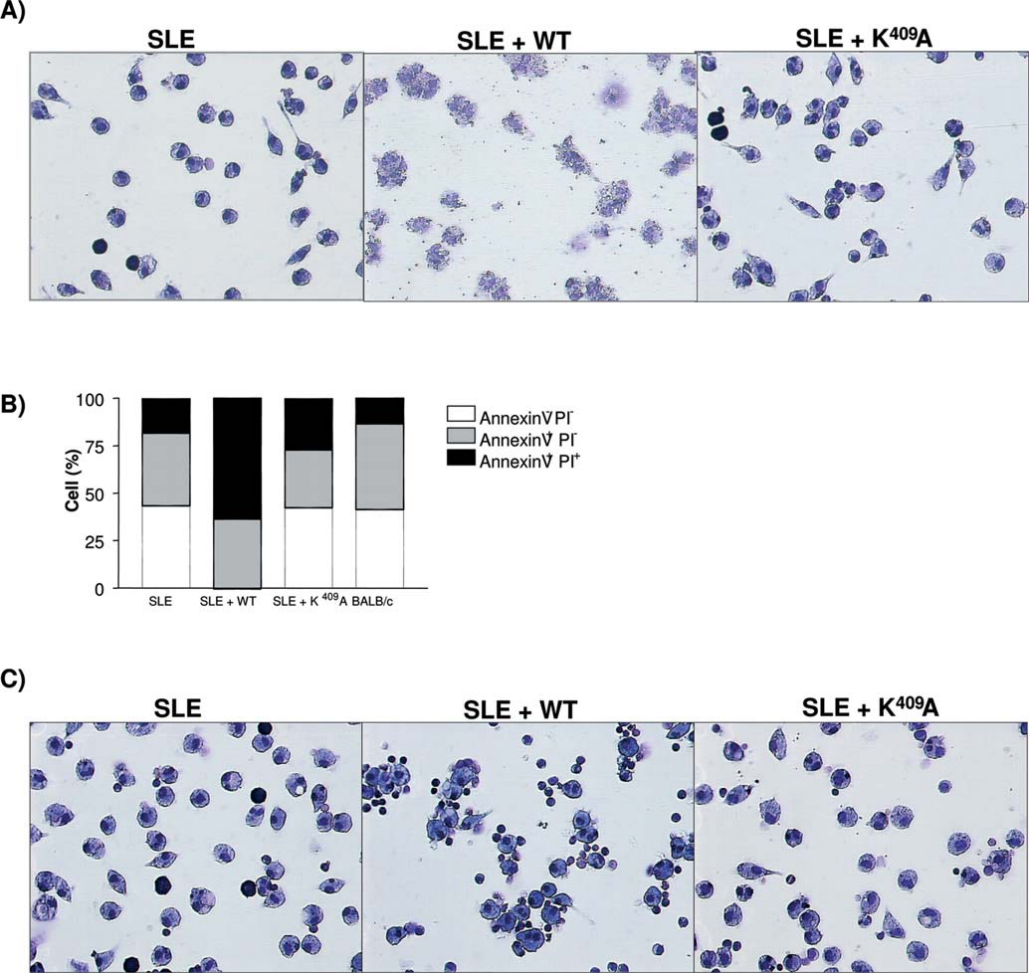
Apoptotic-inducing and cell death effect of treated and untreated [NZBxNZW]F_1_ sera macrophages. BALB/c macrophages were incubated for 3 hours with fresh [A] or heat-inactivated serum [C] from treated or untreated 50 days-old F_1_ mice; light microscopy 100x, HE staining. Percentage of cells binding to annexinV–fluoroisothiocyanate and propidium iodide after 3 hours of incubation with fresh sera [B]. Results are representative of 3 independent experiments.

The role of Hsp65 in increasing the exposition of nuclear components was suggested since there was an increase in anti-DNA IgG2a/IgG1 antibody levels in WT treated [NZBxNZW]F_1_ mice. As reported elsewhere [23], [24] accelerated monocytes/macrophages apoptosis in SLE contributes to impaired clearance of apoptotic cells and amplification of apoptotic material. Taken together, these events lead to the investigation of the apoptosis-inducing effect of WT and K^409^A treated mice sera. BALB/c peritoneal cells were subjected to different sera treatments were analyzed for apoptosis and necrosis. Morphological analysis showed that serum from untreated and K^409^A rHsp65-treated mice had the same effect on macrophages as normal BALB/c serum [Figure 5A]. Quantification of cell death in these samples showed that less than 25% of the cells are annexin^+^PI^+^, which probably refers to late stage apoptosis and not necrosis, since under microscopic analysis we did not observe lysed cells, for the SLE group, as shown in Figure 5A. In contrast, incubation of cells with serum from WT rHsp65 treated mice resulted in necrosis, revealing accumulation of cell *debris* and intense membrane destruction [SLE+WT group - Figure 5A]. Quantification analyses showed that cells with serum from WT treated mice presented 36 and 64% of apoptosis and necrosis, respectively [*p*<0.001], when compared to untreated controls [Figure 5B]. This necrotic effect is reversed by incubation of BALB/c macrophages with heat-inactivated serum, indicating a possible role of complement in cellular damage [SLE+WT group - Figure 5C]. No histological alterations were observed on peritoneal macrophages treated with the purified WT or K^409^A rHsp65, being suggestive of complex processes and pleotropic action induced by the WT *in vivo* administered.

## Discussion

In a genetically heterogeneous population, individuals submitted to the same immunogenic stimulus, facing toxin or infectious processes will present inflammatory and adaptive qualitative and quantitative distinct immune responses. This variability will depend on different genotypes in the population, as well as on the complexes pre– and post–birth environments to which each individual becomes exposed [30]. The main functional traits such as *inflammatory responses*, *antibody production*, *cell mediated immunity and immune tolerance* regardless of its integrated functions are submitted to independent polygenic controls [31]–[34]. Thus, the complex immune network is essentially pleiotropic and the interactions between these innate and acquired functions ensure the multidirectional protection of a natural population. Furthermore, the environmental variances [VE] represent at least 50% of the total phenotypic variances [VP] for each one of the above mentioned characters. Since these immunobiological functions operate simultaneously during a physiopathological process it is expected a huge impact of the VE during a chronic disease. The genetic factors influencing the symptoms of SLE are extensively described in a recent, reviewed in Crow, 2008, exposing the distinct loci intervening on human SLE development, including the HLA alleles associated with the production of autoantibodies. These seem to be related to MHC Class II molecules that promote the expansion of T cells-specific auto-antigen.

In the present study, although the experimental model is limited to one individual, it was possible to expose the elevated degree and the importance of the environmental factors on SLE and the influence of a *M. leprae* rHsp65 protein in the significant increase of mortality, since in this genetically homogeneous model the VP = VE. The results also indicate the possibility of, in restricting the environmental effects, reducing the autoimmune severity or retarding the successive crises that characterize a degenerative chronic process by administering a mutant Hsp65 molecule. The control of expression and the rupture of Hsp65 balance in SLE development were ascertained through the approach of inductive disequilibrium of physiological and immune states by homologous Hsp. The co-existence of potential pathogens kept under control on hosts, for example, might represent one of the major regulators of balanced self-protective and anti-infective immunity and a vital provider to maintenance of health [35]. Naturally occurring subliminal immune responses intervening for maintenance of neutralization and / or equilibrium of biologically active endogenous molecules must be frequent. Under variable and persistent environmental pressures along life, the organism expresses several molecular targets that could be susceptible to a series of autoimmune episodes that due to homeostasis are surmountable. However, unbalance between cells and molecules and the increased expressions of multifunctional proteins such as IL-6, Hsp, TNF, and can unchain chronic, cumulative and irreversible processes of autoimmunities. From this perspective, the disruption of any metabolic balance might have endogenous roots in terms of mechanisms and triggers. In case of an imbalance in regulation such as hyper stimulation by stress or inflammation, autoimmune disease can emerge [35]. Furthermore, changes in self-protective immunity and transforming physiological functions into a deleterious or self-destructive reaction can also result in autoimmune mechanisms. Some studies hypothesize disequilibrium involving the Hsp65 in autoimmune processes by other theories such as the *immunological homunculus* [35]–[37]. Our data show increased anti-Hsp65 antibodies titers in individuals expressing different autoimmunity processes and that in healthy ones the high levels of anti-Hsp65 antibodies would predispose for autoimmune processes.

**Figure 6:**
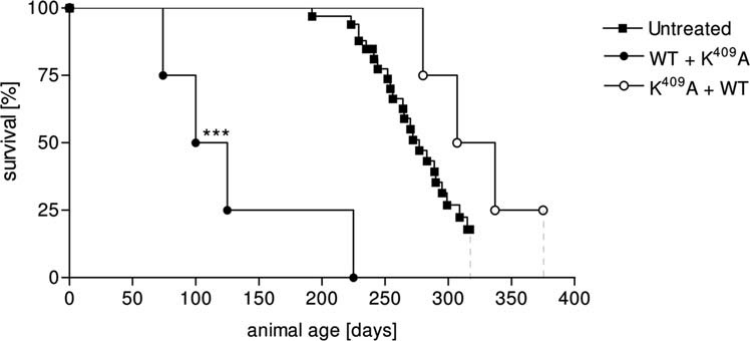
Combined effect of WT and K^409^A Hsp65 in survival time. Forty-five days-old female [NZBxNZW]F_1_ mice were treated with WT or K^409^A and after 7 days, received K^409^A or WT rHsp65. Untreated group has been the reference group for log-rank analysis; ***p<0.001. Results are representative of 2 independent experiments.

The differences in survival time of rHsp65 WT treated groups compared to untreated were significant [*p*<0.05 – Figure 1A]. A positive correlation of 0.81 between time of WT administration and mortality was demonstrated together with the reduction of the VE value contrasting with the VE presented by the untreated group [Figure 1A and 1B]. The untreated animals showed classical clinical murine lupus signs such as anemia, proteinuria and progressive ascite since 6 months–old whereas the WT treated group does not develop any signal even when treated at different ages. The qualitative involvement of the Hsp65 is observed by rHsp65 WT administration in [NZBxNZW]F_1_ mice at different ages. Significant decrease of the mean survival time that was about 50%, when it was administrated at young 45 days-old mice [Figure 1B] was observed. The observation that animals treated with WT present increased 25-fold of anti-DNA Ig2a/IgG1 ratio when compared to untreated mice suggests the possible involvement of the Hsp65 on exposition and amplification of the expression of nuclear antigens in SLE [Figure 2C]. Moreover, there are significant differences in apoptosis and necrosis of cell treated with sera from 50 days-old mice. Sera from WT treated F_1_ mice induced necrosis in BALB/c macrophages. This event can be correlated to the higher and earlier anti-DNA IgG isotypes antibody titers in this group [Figure 5A – group SLE+WT]. Neither the sera from untreated group nor the sera from F_1_ K^409^A treated showed increase in the percentage of annexin^+^PI^+^ cells [Figure 5B]. Progressive cellular apoptosis and necrosis increasing with animal aging was demonstrated by other studies showing that macrophage–lysis is mediated by autoantibodies to Hsp65/60 and identified human Hsp60 epitopes in monocytes [38], [39]. The observation that heat inactivated serum from WT-treated mice reversed the necrotic-induced effect in BALB/c macrophages also suggests a role for complement in cell lysis. The observation that serum from 250 days-old K^409^A treated and untreated mice were effective in inducing necrosis in BALB/c peritoneal cells [data not shown], suggests that this event is related to the disease, rather than a side effect due to Hsp65 treatment. Thus, it seems clear that the WT rHsp65 administration might accelerate antibody response against putative receptors on the cell surface. It has recently been shown that SLE-prone mice as well as SLE patients produce autoantibodies against scavenger receptors expressed on macrophages, affecting apoptotic cellular uptake [40]. Although we did not evaluate the presence of anti-scavenger receptor antibodies, these published data give support to the hypothesis that in our model treatment with the WT Hsp65 protein may enhance the production of these autoantibodies leading to cellular lysis by the classical complement activated pathway. The results of non-responsiveness against Hsp65 WT and the responses to anti-Hsp65 K^409^A of the genetically selected high responder H_III_ mice indicates that the WT molecule is non immunogenic for the susceptible [NZBxNZW]F_1_ mouse whereas the one-point mutated K^409^A rHsp65 molecule seems to be effective in deviating the response towards immunity. Since K^409^A administration had no effect on mortality of [NZBxNZW]F_1_ it was evaluated if K^409^A administration before or after WT injection would inhibit or reverse the effect of the WT protein in accelerating death in these animals. Forty-five days-old mice were treated with K^409^A protein and 7 days later with WT molecule [K^409^A+WT group] and vice-versa. Survival in the former group was significantly enhanced – 294±19 days – when compared to those WT+K^409^A treated group that showed a MST of 100±25 days [Figure 6]. These results show that K^409^A is able to inhibit but not reverse the effect of rHsp65 WT protein on mortality of the [NZBxNZW]F_1_ mouse. These data suggest that the *in vivo* rHsp65 WT effect on cell damages result in complex physio– and immunological processes involving expressions and interactions of other biologically active molecules.

Precocious death in wild type-treated mice may be related to aggravation of vascular lesions. This hypothesis comes from observations that animals that were bled by the retro-orbital plexus clearly showed delay in bleeding interruption compared to the other groups suggesting vascular fragility in WT Hsp65-treated animals. It must be stressed that there is no differences on *in vitro* coagulation when treated and untreated mice were compared; but it is possible that this does not represent what occurs *in vivo* since other factors related to vascular homeostasis may not be reproduced *in vitro*. In the literature, autoantigenic candidates in the development/progression of atherosclerosis include among others, Hsp and β2-glycoprotein-I, being this last a target of anticardiolipin antibodies [41], [42]. Although both, anti-cardiolipin and anti-Hsp60/65 antibodies are enhanced in lupus patients, there are still no evidences if these events occur independently. Anti-Hsp65 antibodies have been found to correlate with progression of carotid atherosclerosis [43] by promoting endothelial cell cytotoxicity [44]. Moreover, the observation that mycobacterial Hsp65 was capable of inducing arteriosclerosis in rabbits after immunization [45] as well as preventing autoimmune atherosclerosis after induction of oral tolerance reinforces the involvement of Hsp65 in this pathology [46]. In infections, development of anti- β2-glycoprotein-I dependent anti-cardiolipin antibodies is restricted to *Mycobacterium leprae* and these antibodies are more pathogenic compared to regular anti-cardiolipin antibodies [47]. Considering that Hsp65 is the major antigen from *Mycobacterium* and that *Mycobacterium leprae* rHsp65 was used in this model, it can be conjectured that anti-cardiolipin and anti-Hsp65 antibodies could be involved in vascular damage and consequently, accelerated death in WT Hsp65-treated mice. Future analysis of the intrinsic immune and physiopathological mechanisms including pro-inflammatory cytokines and the anti-cardiolipin antibodies in the serum of WT- and K^409^A-treated and untreated mice, as well as investigation of vascular lesions, will clarify the role of these soluble molecules, anti-cardiolipin and anti-Hsp antibodies in this model.

Our data clearly showed and enhanced IgG2a/IgG1 anti-DNA antibody ratio in the serum of WT-treated F_1_ compared to the other groups. In mice, IgG2a and IgG2b antibodies are generally considered to be the most potent activators and effectors molecules, able to bind to all of the γ-chain–containing activating Fc receptors [FcγR] *in vitro* [48]. The effector function of IgG1, IgG2a and IgG2b antibodies depend on the threshold for activation of immune cells that can be determined by high or low expression of Fc inhibitory receptors [FcγRIIB] on these cells and the affinity of IgG isotypes to FcγR. In this manner, binding of IgG2a or IgG2b to FcγR and FcγRIIB result in an activating/inhibitory [A/I] ratio of immune cells significantly higher as compared to IgG1antibody [48]. Therefore it would be plausible to expect that acceleration of death in WT-treated F_1_ mice could be in part due to the pro-inflammatory effect of monocyte/macrophage activation mediated by anti-DNA IgG2a autoantibodies. On the other hand, the lack of effect of the K^409^A administration in the mortality of F_1_ animals can be due to the modulation of the anti-DNA IgG2a/IgG1 antibody ratio reflecting in a more balanced activating/inhibitory ratio of immune cells by IgG2a-FcγR engagement. Moreover, as shown in Figure 4, higher IFN-γ levels in the serum of Hsp65 K^409^A treated mice are suggestive of a modulatory effect. In some T cell mediated autoimmune diseases such as rheumatoid arthritis [49] and uveoretinitis [50] IFN-γ has been shown to play a protective role in the development and exacerbation of the disease, respectively. Although speculative, we can raise the hypothesis that, in our model, enhanced production of IFN-γ might suppress the differentiation of Th17 cells or interfere with the Th1/Th2 balance resulting in the modulation of the response. Although mean survival time was not different when compared to untreated mice, variance [VE] was significantly reduced suggesting that the impact of environmental factors in disease progression was neutralized by the treatment. This observation supports a role for IFN-γ in the initial phase of the disease. Moreover it is possible that subsequent injections with the mutant protein could sustain the production of this cytokine throughout life span and ameliorate SLE. There were no differences in glomerulonephritis analyses and seric urea levels between treated or untreated F_1_ groups of animals. All these data denote that although WT Hsp65 treatment enhances anti-DNA production and it does not reflect in any kidney injury.

Finally, returning to Prigogine's principle that dissipative structures keep the ordered state at the cost of irreversibility it is reasonable to assume that the immune system works like one: faster reiterated rounds of stimulation, the system never is able to come back to an original state. Moreover, natural or induced immunizations, as well as autoimmunity processes, lead to distinct stages of innate or acquired responses influenced by endogenous and/or exogenous environmental factors acting from the prenatal period throughout life. The immunological history of an individual is unique and irreversible being cumulative, that is, in the continued aggravation [***a***] of an autoimmune process, there is increase of the progressive entropy [**Δ** *_i_*] that results in inability of tissue regeneration by affected systems. Therefore, the autoimmune syndromes are not reversible in the sense that the original immune state before the beginning of the pathology cannot be reacquired.

A linear equation is suggested for image a distinct chronic degenerative disease of an individual [***y***]:
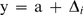



The **Δ** is the increasing entropy [or disorder] and always >0, higher in chronic degenerative disease, and ***i*** represents the number of autoimmune episodes being a time-dependent discrete variable.

In this study, it is clear that the WT and K^409^A molecules cause different disorders: whereas passive administration of WT *M. leprae* Hsp65 lead to rupture of endogenous equilibrium by enhancing the entropy of the immunobiological system, the K^409^A molecule could neutralize this pattern.

In spite of the impossibility of healing, the significantly low VE value in the K^409^A rHsp65 treated individual indicates that a delay occurs in the appearance of symptoms, and the possibility of precocious death in the studied [NZBxNZW]F_1_ individual. Based on these results, it would be possible to think that the employ of point mutated molecules correlated to endogenous, biologically active proteins could be used as therapy, thereby minimizing and retarding the SLE, as well as the emergence of other autoimmune diseases, or reducing the progressive tissue damage and improving life quality.

## Materials and Methods

### Expression of the recombinant *M. leprae* Hsp65 in *Escherichia coli* and purification

Clone pIL161, containing the coding sequence of the *M. leprae* Hsp65, was a gift of Prof. Célio L. Silva from the University of São Paulo, Ribeirão Preto, Brazil, and its mutated form, containing the sequence of the proteolytically non active K^409^A form [17] were amplified in *E. coli* DH5α cells. Expression of the recombinant proteins WT and K^409^A rHsp65 was performed as described in [17]. The recombinant proteins [WT or K^409^A rHsp65] present in the bacterial extract pellet were solubilized with 6 M urea, submitted to preparative SDS-PAGE and elution of the Hsp65 band from the polyacrylamide gel slice was performed to obtain purified WT and K^409^A rHsp65 [51]. The homogeneity of the recombinant *M. leprae* Hsp65 preparations was analyzed by polyacrylamide gel electrophoresis [52] followed by silver staining and submitted to peptide mass fingerprinting analyses, performed as described in [53]. WT and K^409^A rHsp65 protein concentrations were determined as previously described [54].

### Animals

Mice were maintained at the animal facility of the Immunochemistry Laboratory from Butantan Institute and caged and handled under ethical conditions, according to international rules of animal care by the International Animal Welfare Recommendations [55]. New Zealand Black [NZB] female and New Zealand White [NZW] male mice were obtained from the University of São Paulo, Brazil. Parental lines were mated in our Laboratory to produce the [NZBxNZW]F_1_ hybrids. After weaning, the F_1_ hybrids were housed in groups of four-six in plastic cages filled with hardwood bedding, provided with water and rodent chow *ad libitum.* The animals were kept in a room with controlled lighting [12-h light/dark cycle], pressure, humidity and temperature [24±2°C]. Due to the fact that murine lupus is more prevalent in females [56] only this gender was used in the present study. The genetically selected high responder mice [H_III_ line] were bred in the animal facility of the Immunochemistry Laboratory from Butantan Institute [57]. Female H_III_ mice were immunized with WT or K^409^A rHsp65 when 3-month-old.

### Treatment with the WT and K^409^A rHsp65 molecules

Female [NZBxNZW]F_1_ mice at the age of 45, 75 or 125 days were inoculated i.p. with a single dose of 2.5 µg of WT or K^409^A rHsp65 of M. leprae in 0.2 ml of phosphate buffer saline pH7.4 [PBS]. Untreated mice were treated with 0.2 ml of PBS. rHsp65 treated and untreated mice were periodically bled and the individual serum samples stored at −20°C until titration of the anti–DNA and anti–Hsp65 antibodies, which was performed by ELISA. Animals were evaluated during 300 days of age or until their natural death. For the longevity evaluations, mice were periodically examined for clinical signs that include development of ascites, lethargy, anorexia, and death.

### Titration of anti-DNA antibodies

Levels of anti–DNA IgG and the IgG1 and IgG2a isotypes were determined by ELISA. Briefly, 96–well microtiter plates [Nunc MaxiSorp™, Roskilde, Denmark] were coated with 1 µg/well of native salmon sperm DNA [Sigma-Aldrich] diluted in 50 µl of 10 mM TRIS–HCl pH7.5, 1 mM EDTA overnight at 4°C. Plates were washed three times with PBS containing 0.05% Tween 20 [PBS–T] and the wells were blocked with 1% PBS-bovine serum albumin [PBS–BSA] at 37°C for 1h. To each well 100 µl of 1:16 diluted serum sample were added and incubated at 37°C for 1h. Plates were washed again and wells were incubated with peroxidase–labelled anti-mouse IgG [1:2500], IgG1 [1:1000] or IgG2a [1:1000] at 37°C for 1h. After further washing 100 µl of freshly substrate solution [containing 0.5mg/mL *o*–phenylenediamine [Sigma Chemical Company, St. Louis, MO, USA] and 0.03% H_2_O_2_ in citrate/phosphate buffer at pH4.9] was added to each well, and these were then incubated at 37°C until 10 min and the reaction was ended with 50 µl of 0.2 M citric acid. The optical density was measured at λ490nm. The antibody titers were expressed as log_2_ of the reciprocal serum dilution giving an absorbance value of 20% of the saturation level.

### Titration of anti-Hsp65 antibodies

Specific IgG1 and IgG2a isotypes were detected with indirect ELISA. Microplates [Nunc MaxiSorp™] were coated with 1 µg/well of *Mycobacterium leprae* WT rHsp65 diluted in 100 µl of 0.1 M NaHCO_3_ pH9.6, overnight at 4°C. Further steps were performed as described above. Serum was serially diluted starting at 1:16. Anti–Hsp65 antibodies were measured in the serum of WT and K^409^A rHsp65 treated mice. Titers were expressed as log_2_ of the reciprocal serum dilution giving an absorbance value of 20% of the saturation level.

### IFN-γ, TNF-α and IL-6 assays

Levels of IFN-γ, TNF-α and IL-6 were determined in serum of WT or K^409^A rHsp65 treated and untreated [NZBxNZW]F_1_ mice by ELISA in 80 days-old. Cytokine measurements were performed using BD OptEIA enzyme-linked immunosorbent assay sets [BD Biosciences Pharmingen, SanDiego, CA, USA] according to the manufacturer's instructions.

### Renal function and Histology

Blood urea nitrogen [BUN] measurements were performed in duplicate by the urease method. Kidney specimens were fixed in 10% formalin and embedded in paraffin. After processing, 5 µm tissue sections were stained with hematoxylin and eosin [HE] and microscopic analysis was conducted by a trained pathologist.

### Preparation of adherent peritoneal cell cultures

Peritoneal cells were obtained from the peritoneal cavity of non–immunized BALB/c mice. Cells were harvested in the absence of any inflammatory stimulus. Approximately 5 ml of Dulbecco's modified Eagle's medium [DMEM] [Cambrex, Verviers, Belgium] were injected into the peritoneal cavity of the mouse and subsequently collected. Cells were washed and transferred to DMEM supplemented with 10^−5^ M 2-mercaptoethanol [Sigma Chemicals Co, St. Louis, MO, EUA] 2 mM L-glutamine, 0.1 mM vitamins, 1 mM sodium pyruvate, 0.1 mM non-essential amino acids and 100 µg/ml gentamicine all purchased from Gibco BRL [Rockville, NY, USA]. Cells were counted, tested for viability with Trypan blue and incubated [2.5×10^5^ cells] for 4 hours in a 8 well chamber slide [Nalge Nunc International, IL, USA] at 37°C in an atmosphere containing 5% CO_2_. Non-adherent cells were removed by washing the wells with medium. Cells were incubated for 3 hours with 20% serum obtained from female [NZBxNZW]F_1_ treated or not with 2.5 µg of WT or K^409^A rHsp65 [this approach was modified from [58]]. Normal BALB/c mouse serum [NMS] was used as negative control. After incubation period medium was withdrawn and slides were stained with hematoxilin and eosin using the Protocol Hema3 kit [Biochemical Sciences, Swedesboro, NJ].

### Cell death evaluation

Binding of annexinV–fluoroisothiocyanate [FITC] and propidium iodide [PI] [BD Biosciences Pharmingen, SanDiego, CA, USA] to the cells was used to detect viable, early apoptotic and late apoptotic or necrotic cells by flow cytometry. Peritoneal cells [5×10^5^] were cultured in 1 mL of complete medium containing 20% of mouse serum from WT or K^409^A rHsp65 treated [NZBxNZW]F_1_ mice for 16 hours. After incubation cells were washed in annexin buffer [10 mM Hepes, pH7.4, 150 mM NaCl, 5 mM KCl, 1 mM MgCl_2_, 1.8 mM CaCl_2_] and labeled with annexinV–FITC [1:500] in 100 µl of annexin buffer for 20 minutes at room temperature in the dark. Immediately before analysis cells were stained with PI [1:100]. Cells were analyzed in a FACSCalibur [CellQuest software] cell cytometer [BD Biosciences].

### WT and K^409^A rHsp65 combined administration

Female [NZBxNZW]F_1_ mice groups with 45 days-old were inoculated i.p. with a single dose of 2.5 µg of WT or K^409^A rHsp65 of *M. leprae* in 0.2 ml of PBS. pH7.4. Seven days after, they received more 2.5 µg of the opposite Hsp, i.p. WT+K^409^A and K^409^A+WT groups were periodically bled; individual serum sample titration and clinical signs were evaluated according to describe above.

### Statistical analysis

All data are expressed as the mean [X] and standard deviation [SD] or environmental variance [VE]. Statistical significance was set at *p*<0.05 by the Unpaired t–test. The Kaplan-Meier plot for survival was analyzed by log-rank test. The correlation coefficients were determined by the linear regression of group data comparing the mean survival time [MST] with age, dose or administration period of WT or K^409^A rHsp65.

## References

[1] Lindquist S (1986). The heat-shock response.. Annu Rev Biochem.

[2] Winfield JB (1989). Stress proteins, arthritis, and autoimmunity.. Arthritis Rheum.

[3] Jones DB, Hunter NR, Duff GW (1990). Heat-shock protein 65 as a beta cell antigen of insulin-dependent diabetes.. Lancet.

[4] Young RA (1990). Stress proteins and immunology.. Annu Rev Immunol.

[5] Young RA, Elliott TJ (1989). Stress proteins, infection, and immune surveillance.. Cell.

[6] Thole J, van Der Zee R, McFadden J (1990). The 65kD antigen: molecular studies on an ubiquitous antigen.. Molecular Biology of the Mycobacteria.

[7] Young D, Lathigra R, Hendrix R, Sweetser D, Young RA (1988). Stress proteins are immune targets in leprosy and tuberculosis.. Proc Natl Acad Sci U S A.

[8] Cohen IR (1991). Autoimmunity to chaperonins in the pathogenesis of arthritis and diabetes.. Annu Rev Immunol.

[9] Kohm AP, Fuller KG, Miller SD (2003). Mimicking the way to autoimmunity: an evolving theory of sequence and structural homology.. Trends Microbiol.

[10] Oldstone MB (1987). Molecular mimicry and autoimmune disease.. Cell.

[11] Wick G, Perschinka H, Millonig G (2001). Atherosclerosis as an autoimmune disease: an update.. Trends Immunol.

[12] Pockley AG, Shepherd J, Corton JM (1998). Detection of heat shock protein 70 (Hsp70) and anti-Hsp70 antibodies in the serum of normal individuals.. Immunol Invest.

[13] Stephanou A, Latchman DS, Isenberg DA (1998). The regulation of heat shock proteins and their role in systemic lupus erythematosus.. Semin Arthritis Rheum.

[14] Pockley AG, Bulmer J, Hanks BM, Wright BH (1999). Identification of human heat shock protein 60 (Hsp60) and anti-Hsp60 antibodies in the peripheral circulation of normal individuals.. Cell Stress Chaperones.

[15] Rea IM, McNerlan S, Pockley AG (2001). Serum heat shock protein and anti-heat shock protein antibody levels in aging.. Exp Gerontol.

[16] Xu Q, Schett G, Perschinka H, Mayr M, Egger G (2000). Serum soluble heat shock protein 60 is elevated in subjects with atherosclerosis in a general population.. Circulation.

[17] Portaro FC, Hayashi MA, De Arauz LJ, Palma MS, Assakura MT (2002). The Mycobacterium leprae hsp65 displays proteolytic activity. Mutagenesis studies indicate that the M. leprae hsp65 proteolytic activity is catalytically related to the HslVU protease.. Biochemistry.

[18] Tsao BP (2003). The genetics of human systemic lupus erythematosus.. Trends Immunol.

[19] Burnet FM, Holmes MC (1965). The natural history of the NZB/NZW F1 hybrid mouse: a laboratory model of systemic lupus erythematosus.. Australas Ann Med.

[20] Theofilopoulos AN, Dixon FJ (1985). Murine models of systemic lupus erythematosus.. Adv Immunol.

[21] Hahn BH (1998). Antibodies to DNA.. N Engl J Med.

[22] Pisetsky DS (1998). Antibody responses to DNA in normal immunity and aberrant immunity.. Clin Diagn Lab Immunol.

[23] Kaplan MJ (2004). Apoptosis in systemic lupus erythematosus.. Clin Immunol.

[24] Kaplan MJ, Lewis EE, Shelden EA, Somers E, Pavlic R (2002). The apoptotic ligands TRAIL, TWEAK, and Fas ligand mediate monocyte death induced by autologous lupus T cells.. J Immunol.

[25] Ishikawa S, Akakura S, Abe M, Terashima K, Chijiiwa K (1998). A subset of CD4+ T cells expressing early activation antigen CD69 in murine lupus: possible abnormal regulatory role for cytokine imbalance.. J Immunol.

[26] Putterman C, Naparstek Y, Cohen IR, Miller A (1994). Murine Models of Spontaneous Systemic Lupus Erythematosus.. Autoimmune Models: A Guidebook.

[27] Struhar D, Harbeck R, Cherniack R (1988). Elastic properties of the excised lungs of NZB/W mice and their correlation with histopathologic changes.. Lung.

[28] Prigogine I (1978). Time, Structure, and Fluctuations.. Science.

[29] Prigogine I (2003). Chemical kinetics and dynamics.. Ann N Y Acad Sci.

[30] Mouton D, Sant'anna OA, Biozzi G (1988). Multigenic Control of Specific and Non-specific Immunity in Mice. A Review.. Livestock Production Sicence.

[31] Biozzi G, Mouton D, Sant'Anna OA, Passos HC, Gennari M (1979). Genetics of immunoresponsiveness to natural antigens in the mouse.. Curr Top Microbiol Immunol.

[32] da Silva AC, de Souza KW, Machado RC, da Silva MF, Sant'Anna OA (1998). Genetics of immunological tolerance: I. Bidirectional selective breeding of mice for oral tolerance.. Res Immunol.

[33] Ibanez OM, Stiffel C, Ribeiro OG, Cabrera WK, Massa S (1992). Genetics of nonspecific immunity: I. Bidirectional selective breeding of lines of mice endowed with maximal or minimal inflammatory responsiveness.. Eur J Immunol.

[34] Sant'Anna OA, Ferreira VC, Reis MH, Gennari M, Ibanez OM (1982). Genetic parameters of the polygenic regulation of antibody responsiveness to flagellar and somatic antigens of salmonellae.. J Immunogenet.

[35] Prohaszka Z, Fust G (2004). Immunological aspects of heat-shock proteins-the optimum stress of life.. Mol Immunol.

[36] Poletaev A, Osipenko L (2003). General network of natural autoantibodies as immunological homunculus (Immunculus).. Autoimmun Rev.

[37] Cohen IR (2007). Biomarkers, self-antigens and the immunological homunculus.. J Autoimmun.

[38] Habich C, Kempe K, Burkart V, Van Der Zee R, Lillicrap M (2004). Identification of the heat shock protein 60 epitope involved in receptor binding on macrophages.. FEBS Lett.

[39] Schett G, Metzler B, Mayr M, Amberger A, Niederwieser D (1997). Macrophage-lysis mediated by autoantibodies to heat shock protein 65/60.. Atherosclerosis.

[40] Wermeling F, Chen Y, Pikkarainen T, Scheynius A, Winqvist O (2007). Class A scavenger receptors regulate tolerance against apoptotic cells, and autoantibodies against these receptors are predictive of systemic lupus.. J Exp Med.

[41] Vaarala O, Alfthan G, Jauhiainen M, Leirisalo-Repo M, Aho K (1993). Crossreaction between antibodies to oxidised low-density lipoprotein and to cardiolipin in systemic lupus erythematosus.. Lancet.

[42] George J, Harats D, Gilburd B, Afek A, Levy Y (1999). Immunolocalization of beta2-glycoprotein I (apolipoprotein H) to human atherosclerotic plaques: potential implications for lesion progression.. Circulation.

[43] Xu Q, Willeit J, Marosi M, Kleindienst R, Oberhollenzer F (1993). Association of serum antibodies to heat-shock protein 65 with carotid atherosclerosis.. Lancet.

[44] Schett G, Xu Q, Amberger A, Van der Zee R, Recheis H (1995). Autoantibodies against heat shock protein 60 mediate endothelial cytotoxicity.. J Clin Invest.

[45] Xu Q, Dietrich H, Steiner HJ, Gown AM, Schoel B (1992). Induction of arteriosclerosis in normocholesterolemic rabbits by immunization with heat shock protein 65.. Arterioscler Thromb.

[46] Harats D, Yacov N, Gilburd B, Shoenfeld Y, George J (2002). Oral tolerance with heat shock protein 65 attenuates Mycobacterium tuberculosis-induced and high-fat-diet-driven atherosclerotic lesions.. J Am Coll Cardiol.

[47] Hojnik M, Gilburd B, Ziporen L, Blank M, Tomer Y (1994). Anticardiolipin antibodies in infections are heterogenous in their dependency on beta 2-glycoprotein I: analysis of anticardiolipin antibodies in leprosy.. Lupus.

[48] Nimmerjahn F, Ravetch JV (2006). Fcgamma receptors: old friends and new family members.. Immunity.

[49] Kim EY, Chi HH, Bouziane M, Gaur A, Moudgil KD (2008). Regulation of autoimmune arthritis by the pro-inflammatory cytokine interferon-gamma.. Clin Immunol.

[50] Yoshimura T, Sonoda KH, Miyazaki Y, Iwakura Y, Ishibashi T (2008). Differential roles for IFN-gamma and IL-17 in experimental autoimmune uveoretinitis.. Int Immunol.

[51] Pereira CM, Sattlegger E, Jiang HY, Longo BM, Jaqueta CB (2005). IMPACT, a protein preferentially expressed in the mouse brain, binds GCN1 and inhibits GCN2 activation.. J Biol Chem.

[52] Laemmli UK (1970). Cleavage of structural proteins during the assembly of the head of bacteriophage T4.. Nature.

[53] Westermeier R, Naven T (2002). Proteomics in Practice: A Laboratory Manual of Proteome Analysis..

[54] Bradford MM (1976). A rapid and sensitive method for the quantitation of microgram quantities of protein utilizing the principle of protein-dye binding.. Anal Biochem.

[55] Giles AR (1987). Guidelines for the use of animals in biomedical research.. Thromb Haemost.

[56] Helyer BJ, Howie JB (1963). Spontaneous auto-immune disease in NZB/BL mice.. Br J Haematol.

[57] Siqueira M, Bandieri A, Reis MS, Sant'anna OA, Biozzi G (1976). Selective breeding of mice for antibody responsiveness to flagellar and somatic antigens of salmonellae.. Eur J Immunol.

[58] Bengtsson AA, Sturfelt G, Gullstrand B, Truedsson L (2004). Induction of apoptosis in monocytes and lymphocytes by serum from patients with systemic lupus erythematosus - an additional mechanism to increased autoantigen load?. Clin Exp Immunol.

